# Comparative Analysis of Morphology and Targeted Fatty Acid Profiling of Adipose Tissues from Different Depots in the Junggar Bactrian Camel

**DOI:** 10.3390/ani16152412

**Published:** 2026-08-05

**Authors:** Xiaokang Chang, Yi Su, Manjun Zhai, Jun Meng, Yaqi Zeng, Jianwen Wang, Wanlu Ren, Xinkui Yao

**Affiliations:** 1 College of Animal Science, Xinjiang Agricultural University, Urumqi 830052, China; changxk312@126.com (X.C.); 13335339131@163.com (Y.S.); zhaimanjun@yeah.net (M.Z.); mengjun@xjau.edu.cn (J.M.); zengyaqi@xjau.edu.cn (Y.Z.); dkwjw@xjau.edu.cn (J.W.); renwanlu@xjau.edu.cn (W.R.); 2 Xinjiang Key Laboratory of Equine Breeding and Exercise Physiology, Urumqi 830052, China

**Keywords:** Junggar Bactrian camel, adipose tissue, morphology, targeted fatty acid profiling, fatty acids

## Abstract

Adipose tissue exhibits profound anatomical heterogeneity that underpins distinct physiological roles, yet depot-specific morphological and metabolic differentiation in camelids remains elusive. Here, we systematically characterized hump adipose tissue (HAT), mesenteric adipose tissue (MAT), pericardial adipose tissue (PAT), and perirenal adipose tissue (PRAT) from 13 adult male Junggar Bactrian camels using H&E histomorphometry and GC-MS-based targeted fatty acid profiling. Morphologically, PRAT displayed significantly larger mean adipocyte equivalent diameter and cross-sectional area than HAT and MAT (*p* < 0.05); conversely, MAT exhibited the highest adipocyte density, whereas HAT featured smaller, uniformly sized cells with densely packed lipid droplets. Targeted fatty acid profiling revealed that HAT contained markedly elevated monounsaturated fatty acids—particularly palmitoleic acid (C16:1 n-7) and oleic acid (C18:1 n-9)—constituting 34.60% ± 2.82% of total fatty acids compared to 28.32% ± 1.23% in visceral depots (*p* < 0.01). In contrast, visceral fats (MAT, PRAT, PAT) were dominated by saturated fatty acids, with MAT exhibiting the highest stearic acid (C18:0) content. Principal component analysis and partial least squares discriminant analysis (PLS-DA) revealed marked metabolic differentiation of HAT from other depots, while PAT and PRAT shared highly similar profiles. Differentially abundant metabolites were predominantly enriched in fatty acid biosynthesis and unsaturated fatty acid biosynthesis pathways. These findings reveal distinct depot-associated differences in adipocyte morphology and fatty acid composition. The unique phenotypic profile of HAT is consistent with a putative role in lipid storage, potentially reflecting an adaptation to arid environments, while visceral depots exhibited traits conducive to biomechanical cushioning and metabolic regulation. This work describes depot-associated differences in adipocyte morphology and fatty acid composition in the Junggar Bactrian camel, supporting the potential for valorizing unique camel fat resources.

## Simple Summary

Adipose tissue functions vary by anatomical location, yet depot-specific characteristics in camels remain poorly defined. Here, we integrated histomorphological analysis and targeted fatty acid profiling to examine four major fat depots—hump (HAT), mesenteric (MAT), pericardial (PAT), and perirenal (PRAT)—in adult male Junggar Bactrian camels. We found that HAT is characterized by small, densely packed adipocytes and a distinct lipid profile featuring elevated monounsaturated fatty acids (e.g., oleic and palmitoleic acids). In contrast, visceral depots exhibited larger adipocyte morphology and higher proportions of saturated fatty acids. These findings delineate distinct depot-dependent morphological and metabolic profiles, suggesting that HAT may serve as a specialized energy reservoir, whereas visceral depots likely fulfill structural and protective roles. This study provides a foundational atlas of adipose specialization in camels, informing future physiological investigations and highlighting the unique compositional traits of camel fat as a potential nutritional resource.

## 1. Introduction

Adipose tissue serves not only as the body’s primary energy reservoir but also as a crucial endocrine organ, exhibiting remarkable structural and functional heterogeneity across distinct anatomical depots [1,2]. Subcutaneous adipose tissue is predominantly involved in lipid metabolism, demonstrating more active substrate utilization, energy turnover, and lipid cycling [3,4]. In contrast, visceral adipose tissue is primarily associated with inflammatory and immune-related functions; its dysregulation can trigger chronic inflammation and insulin resistance through mechanisms such as metabolic reprogramming [5,6]. For instance, pericardial and subcutaneous adipose tissues display significant differences in signaling pathways related to fatty acid oxidation, glycolysis, thermogenesis, and extracellular matrix interaction, with subcutaneous depots exhibiting higher metabolic activity while pericardial fat leans toward structural regulation [7]. Moreover, adipose tissue plays a pivotal role in maintaining systemic metabolic and immune homeostasis via secretion of leptin, adiponectin, and other adipokines, as well as by modulating macrophage M1/M2 polarization balance [8].

The Junggar Bactrian camel (*Camelus bactrianus*) is an economically important livestock species inhabiting the desert regions of northwestern China. Through prolonged natural selection, it has evolved exceptional tolerance to extreme environments and robust immune homeostasis [9,10,11]. The hump adipose tissue (HAT) of Bactrian camels serves as a specialized energy storage organ, enabling sustained endurance for weeks under food-scarce desert conditions [12,13]. While the fundamental biochemical pathways governing adipocyte differentiation and lipid metabolism are evolutionarily conserved across mammals, their physiological manifestations in camels reflect unique adaptations to extreme aridity and temperature fluctuations. Unlike other species, camels rely on the dorsal hump primarily for energy storage. Beyond the hump, however, widely distributed depots such as pericardial (PAT), perirenal (PRAT), and mesenteric (MAT) adipose tissues play crucial roles in protecting visceral organs and maintaining homeostasis. Despite their recognized presence, the functional specialization and specific metabolic signatures distinguishing these depots have not been fully elucidated.

Recent advances in multi-omics technologies have begun to unravel the metabolic complexity of camel adipose tissue. Comparative transcriptomic analysis of three distinct depots in Bactrian camels revealed that hump adipose tissue (HAT) exhibits a superior regulatory capacity for triglyceride storage and mobilization compared to the greater omentum [14]. Furthermore, lipid compositional studies indicate that while the fatty acid profiles are largely conserved between the hump and abdominal adipose tissues, saturated fatty acids predominate over their unsaturated counterparts [15]. In contrast, investigations into muscle tissues—focusing on meat quality traits—have been more extensive. Proteomic and metabolomic profiling across different ages and muscle groups highlights that camel meat is characterized by high protein content, low fat levels, and abundant polyunsaturated fatty acids, with an amino acid composition aligning with FAO/WHO ideal protein standards [16]. Subsequent studies have further elucidated the proteome and lipidome of various muscle depots to decipher the mechanisms underlying meat quality variation [17,18]. However, despite these foundational efforts, a critical knowledge gap remains: systematic comparative analyses targeting the intrinsic molecular heterogeneity among distinct camel adipose depots remain conspicuously absent.

Targeted fatty acid profiling enables precise quantification of specific metabolites within key metabolic pathways, thereby offering a more accurate depiction of the metabolic status and biochemical signatures of different depots [19]. Therefore, the present study aims to systematically compare four adipose depots (HAT, MAT, PAT, and PRAT) from Junggar Bactrian camels using targeted fatty acid profiling combined with histomorphological observation. By delineating the convergent and divergent features of fatty acid repertoires and tissue microarchitecture, this study establishes a foundational molecular blueprint of depot-specific functionality, offering theoretical insights into the metabolic specialization of adipose tissues in desert-adapted mammals.

## 2. Materials and Methods

### 2.1. Experimental Animals and Sample Collection

This study was approved by the Animal Welfare and Ethics Committee of Xinjiang Agricultural University (approval number: 2025032). Importantly, the camels were not slaughtered exclusively for this research; tissue sampling was conducted as a byproduct of routine commercial slaughter at a government-inspected abattoir, complying with the National Standard for Humane Slaughter of Livestock. Thirteen clinically healthy, 5- to 6-year-old male Junggar Bactrian camels with an average body weight of 460 ± 25 kg were selected for this study. All animals were housed under a uniform intensive fattening system at the same pasture in the Junggar Basin, Xinjiang, China. Throughout the rearing period, camels were allowed to graze freely during daylight hours and were supplemented daily with a standard concentrate diet (corn-soybean meal-based) and alfalfa hay ad libitum, with continuous access to water. To standardize metabolic status prior to sampling, all animals were fasted for 12 h but allowed free access to water before slaughter.

Following exsanguination, four distinct adipose depots—hump adipose tissue (HAT), mesenteric adipose tissue (MAT), perirenal adipose tissue (PRAT), and pericardial adipose tissue (PAT)—were rapidly harvested from each of the 13 individuals. Samples designated for histomorphological analysis were immediately fixed in 4% paraformaldehyde, whereas those intended for targeted fatty acid profiling were snap-frozen in liquid nitrogen and stored at −80 °C until further analysis. Given that all four tissue types were collected from the same individuals, the camel was considered the experimental unit (*n* = 13), and the adipose depot was treated as a within-animal variable. This paired experimental design was employed to control for inter-individual genetic and environmental variability.

### 2.2. Histomorphological Analysis

Paraformaldehyde-fixed adipose samples were dehydrated through a graded ethanol series (75%, 85%, 90%, 95%, and 100%), cleared with xylene, embedded in paraffin, and sectioned transversely at a thickness of 5 μm using a rotary microtome. Tissue sections were stained with hematoxylin and eosin (H&E) and photographed using an optical microscope (Nikon Eclipse E100, Nikon Corporation, Tokyo, Japan) equipped with a camera system [20].

To ensure unbiased quantification, image analysis was performed by an investigator blinded to the depot identities. For each section, 10 random, non-overlapping fields of view (FOVs) were captured at 20× magnification. Within these FOVs, a minimum of 100 intact adipocytes were manually outlined and measured per sample. Adipocytes were included in the analysis only if they exhibited a spherical morphology with distinct cellular boundaries; cells intersecting the upper or left borders of the image frame, or those exhibiting artifacts (e.g., tearing, folding, or nuclear pyknosis), were systematically excluded to prevent overestimation errors.

We acknowledge the potential for heterogeneity in adipocyte size distribution; however, preliminary saturation analysis indicated that measuring 100 cells across 10 FOVs per section reached a plateau in mean diameter calculation, confirming the adequacy of our sampling strategy. Morphometric parameters, including mean equivalent diameter and mean cross-sectional area, were automatically computed using ImageJ software (version 1.53k, NIH, Bethesda, MD, USA).

### 2.3. Fatty Acid Extraction and Derivatization

Frozen adipose tissues were thawed at 4 °C. Exactly 50 mg of tissue was weighed and homogenized in 5 mL of ice-cold methylene dichloride-methanol (2:1, *v*/*v*). The mixture was vortexed and ultrasonicated at low temperature for 30 min. Following the addition of 2 mL of ultrapure water, the lower organic phase was collected and dried under nitrogen. The residue was reconstituted in 2 mL of n-hexane containing the internal standard, Nonadecanoic acid (C19:0). Methylation was performed at room temperature for 0.5 h. After adding 2 mL of ultrapure water and vortexing, 1000 µL of the upper hexane layer was collected, dried under nitrogen, and re-dissolved in n-hexane for analysis [19].

### 2.4. GC-MS Analysis

Fatty acid methyl esters (FAMEs) were analyzed using an Agilent 7890B GC system coupled with a 5977B MSD mass spectrometer. Separation was achieved on a DB-23 capillary column (60 m × 250 μm × 0.15 μm). The injection volume was 1 µL in splitless mode. The oven temperature was programmed as follows: initial temperature 80 °C; ramped at 20 °C/min to 180 °C (held for 8 min); then ramped at 5 °C/min to 280 °C (held for 3 min). Helium was used as the carrier gas at a flow rate of 1.0 mL/min. A solvent delay of 3 min was employed to protect the filament. The MS operated in electron impact ionization (EI) mode (70 eV) with the ion source at 230 °C and the transfer line at 250 °C. Detection was performed in Scan/SIM mode over a mass range of *m*/*z* 50–550. Specific SIM ions (quantifier and qualifier ions) were monitored for each target analyte to ensure selectivity, with dwell times automatically optimized by the acquisition software. Compound identification was based on a combination of (i) retention time matching against authentic reference standards (Nu-Chek Prep, Inc., Elysian, MN, USA), with a tolerance of <0.1 min, and (ii) mass spectral confirmation, ensuring the sample spectra and ion ratios matched those of the reference standards within a 5% tolerance.

### 2.5. Quality Control and Batch Monitoring

To monitor instrument stability and minimize potential batch effects, a pooled Quality Control (QC) sample, prepared by mixing aliquots from all study samples, was injected after every 10 biological samples throughout the analytical queue. The relative standard deviation (RSD) of the QC samples was maintained below 30%, confirming the robustness and reliability of the dataset for comparative analysis across the four adipose depots.

### 2.6. Data Processing and Quantification

Absolute quantification was performed using seven-point calibration curves, with C19:0 (Nonadecanoic acid) serving as the internal standard for normalization. All standard curves demonstrated a coefficient of determination (R^2^ > 0.99). The limit of detection (LOD) was 0.0004 mg/mL and the limit of quantification (LOQ) was 0.002 mg/mL. Sample concentrations were calculated using the formula C_calc_ × V/m, where C_calc_ is the concentration derived from the standard curve, Vis the extraction volume, and mis the tissue mass. Total saturated fatty acids (SFA), monounsaturated fatty acids (MUFA), and polyunsaturated fatty acids (PUFA) were calculated by summing the absolute concentrations of their respective constituent fatty acids. Fatty acid absolute concentrations (μg/g tissue) were quantified via GC-MS. For statistical analyses, these values were converted to relative molar percentages by normalizing to the sum of all quantified FAMEs. Table S1 reports the relative molar percentages of individual fatty acids, calculated by normalizing the absolute concentration of each fatty acid to the sum of all 40 quantified FAMEs.

### 2.7. Differential Abundant Metabolite Identification

Differentially abundant metabolites were identified using a paired framework (four depots harvested from each of the 13 camels). Significance was defined as FDR-adjusted *p* < 0.05 combined with a fold change (FC) cutoff of ≥1.2 or ≤0.83 (equivalent to |log_2_FC| ≥ 0.26), a threshold chosen to balance sensitivity to biologically meaningful shifts with exclusion of technical noise in targeted fatty acid profiling.

### 2.8. Statistical Analysis and Visualization

One-way repeated-measures ANOVA (RM ANOVA, SPSS 26.0) was performed exclusively on adipocyte morphological parameters (mean equivalent diameter; mean cross-sectional area; adipocyte density). Individual camel was set as the subject variable and depot as the repeated-measures factor. Normality (Shapiro–Wilk) and sphericity (Mauchly’s test) were verified; Greenhouse–Geisser correction was applied for sphericity violations. Post hoc pairwise comparisons used Tukey’s HSD test, with Benjamini–Hochberg FDR correction defining significance as FDR-adjusted *p* < 0.05. Bar charts were generated in GraphPad Prism 9.5.0. For the targeted fatty acid profiling data, the same paired experimental design was employed. One-way repeated-measures ANOVA (RM ANOVA) was similarly applied to the relative percentages of individual fatty acids and total fatty acid classes (SFA, MUFA, PUFA), with individual camel treated as the subject variable and adipose depot as the repeated-measures factor. Normality and sphericity were assessed as described above. Post hoc pairwise comparisons were performed using Tukey’s HSD test, followed by Benjamini–Hochberg FDR correction to account for multiple comparisons across the 40 fatty acids.

Multivariate analyses were performed on the Metware Cloud platform (https://cloud.metware.cn). No missing values were detected in the final dataset, and Pareto scaling was applied to balance the contributions of low- and high-abundance metabolites prior to modeling. Unsupervised principal component analysis (PCA) was conducted to visualize global fatty acid profile variations across depots. Supervised partial least squares discriminant analysis (PLS-DA) was further employed to resolve depot-specific fatty acid signatures, with model robustness evaluated via 7-fold cross-validation (optimized for *n* = 13 biological replicates) to derive R^2^X (variance explained in fatty acid data), R^2^Y (variance explained in depot class labels), and Q^2^ (model predictive capacity). Consistent with the empirical results obtained from this cohort, the PLS-DA models yielded R^2^X > 0.6, R^2^Y > 0.4, and Q^2^ > 0.2. While these values are modest compared to larger-scale metabolomic studies, they are acceptable given the moderate sample size (*n* = 13) and the inherent biological variability among individual animals. Two hundred-cycle permutation testing was additionally performed to guard against overfitting, with all models exhibiting Q^2^ intercepts below zero and permutation *p* < 0.05, confirming model validity. PCA score plots and PLS-DA score plots were generated on the same platform.

Pathway enrichment was performed by Metware Biotechnology Co., Ltd. (Wuhan, China) using the Metware Cloud platform. Annotated metabolites were mapped to the Kyoto Encyclopedia of Genes and Genomes (KEGG) Pathway Database (release 109.0, accessed 14 February 2025; https://www.kegg.jp/). The background metabolite set consisted of all 56 fatty acids (short-, medium-, and long-chain) quantitatively profiled in this targeted panel, consistent with the experimental detection scope. Enrichment significance was evaluated via a two-sided hypergeometric test, with multiple comparisons corrected using the Benjamini–Hochberg false discovery rate (FDR) method. Pathways with FDR-adjusted *p* < 0.05 were considered significantly enriched.

To assess the spatial congruence between depot-specific fatty acid compositions and adipocyte morphological characteristics, a Mantel test was performed using the Metware Cloud platform. Two distance matrices were constructed based on 52 samples (13 camels × 4 depots): (1) a Bray–Curtis dissimilarity matrix based on the relative abundances of 40 fatty acids (Pareto-scaled); and (2) a Euclidean distance matrix based on three standardized phenotypic variables (mean equivalent diameter, adipocyte density, and mean cross-sectional area).

## 3. Results and Analysis

### 3.1. Morphology of Adipose Tissues from Different Depots

**Figure 1:**
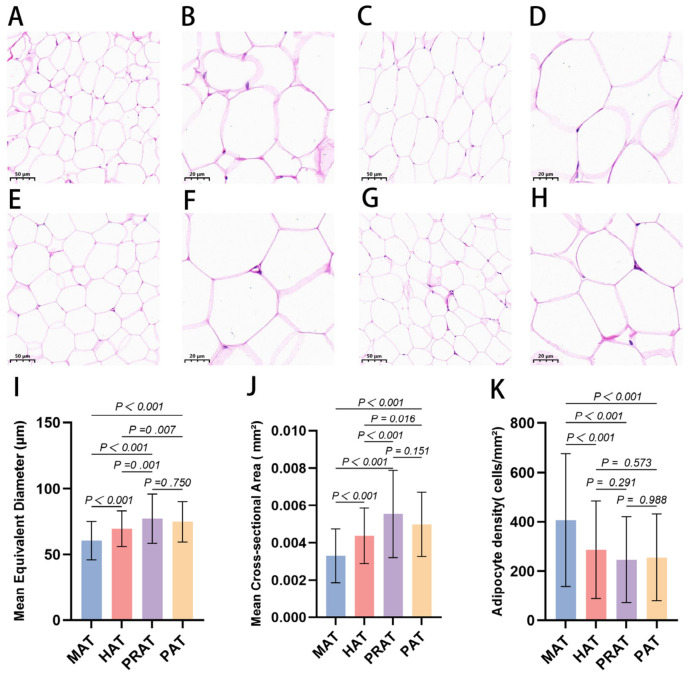
H&E staining and quantitative morphological analysis of adipose tissues from different depots. (**A**) Mesenteric Adipose Tissue (20×, scale bar = 50 μm); (**B**) Mesenteric Adipose Tissue (50×, scale bar = 20 μm); (**C**) Hump Adipose Tissue (20×, scale bar = 50 μm); (**D**) Hump Adipose Tissue (50×, scale bar = 20 μm); (**E**) Perirenal Adipose Tissue (20×, scale bar = 50 μm); (**F**) Perirenal Adipose Tissue (50×, scale bar = 20 μm); (**G**) Pericardial Adipose Tissue (20×, scale bar = 50 μm); (**H**) Pericardial Adipose Tissue (50×, scale bar = 20 μm); (**I**) Mean Equivalent Diameter; (**J**) Mean Cross-sectional Area; (**K**) Adipocyte density.

H&E staining revealed that camel adipocytes exhibited red cytoplasm and blue nuclei with clearly defined cell boundaries (Figure 1A–H). Cells appeared polygonal or rounded. Adipocytes exhibited extensive unstained vacuoles (representing extracted lipids during processing) displacing the nuclei to the cell periphery. Hump adipose tissue (HAT) displayed relatively small and uniformly distributed adipocytes. Mesenteric adipose tissue (MAT) showed variable cell sizes, though overall cell volume remained small. Compared with HAT and MAT, pericardial adipose tissue (PAT) exhibited a marked increase in cell size. Perirenal adipose tissue (PRAT) showed substantially enlarged cell volumes with considerable variation in cell diameter.

Quantitative morphometric analysis determined the mean equivalent diameter, cross-sectional area, and adipocyte density across the four depots (Figure 1I–K and Table 1). PRAT exhibited significantly greater mean equivalent diameter (77.21 ± 18.831 μm) and cross-sectional area (0.0055 ± 0.0023 mm^2^) than both MAT (60.51 ± 14.591 μm and 0.0033 ± 0.0014 mm^2^) and HAT (69.57 ± 13.48 μm and 0.0044 ± 0.0015 mm^2^, *p* < 0.05), although PRAT had a larger mean equivalent diameter and cross-sectional area than PAT (74.88 ± 15.387 μm and 0.0050 ± 0.0017 mm^2^), the difference did not reach statistical significance (*p* > 0.05). PRAT exhibited a significantly lower adipocyte density (246.32 ± 174.946 cells/mm^2^) compared to MAT (407.08 ± 268.928 cells/mm^2^, *p* < 0.05).

### 3.2. Multivariate Analysis of Adipose Tissue Metabolic Profiles

**Figure 2:**
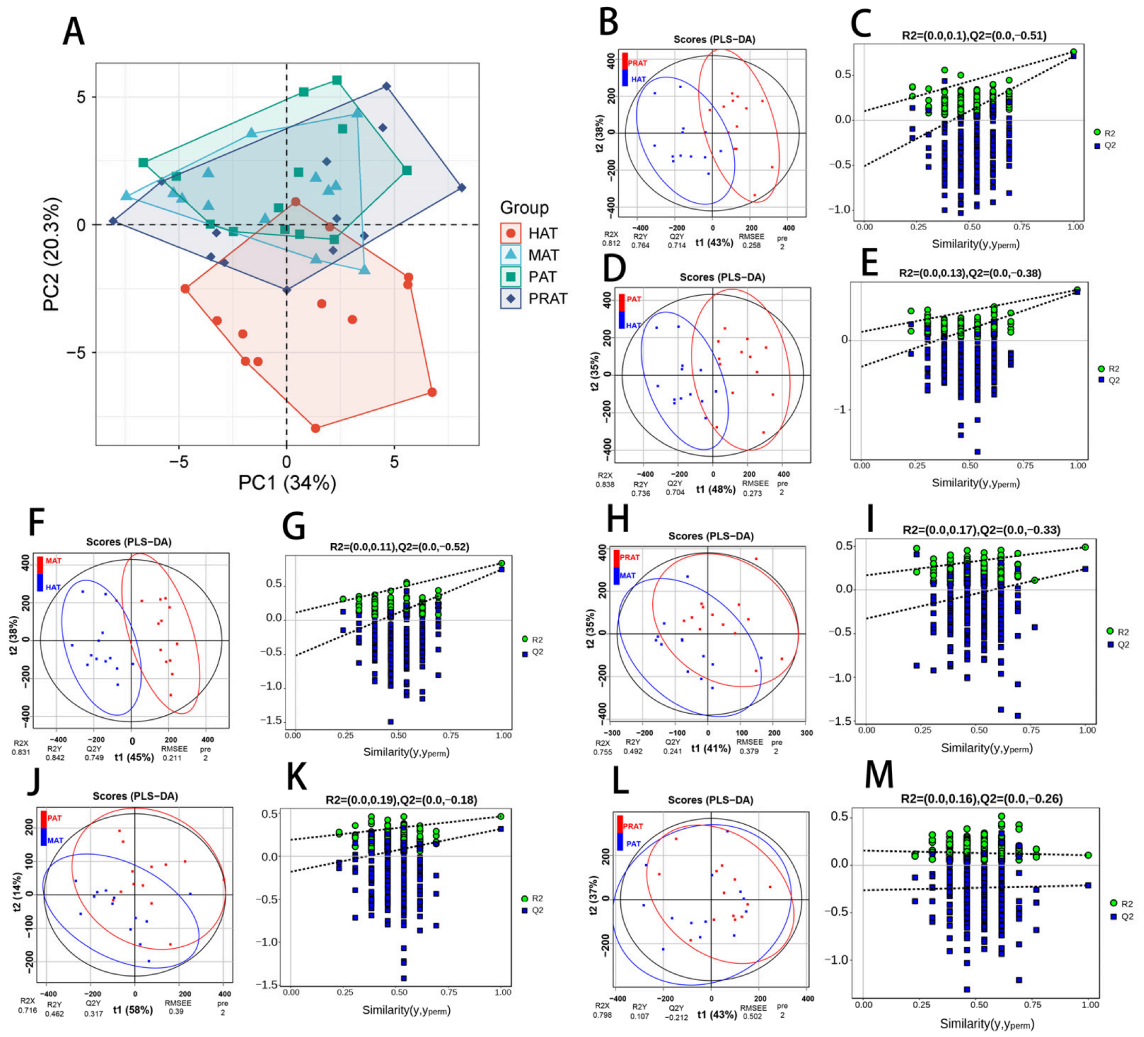
Principal component analysis and partial least squares discriminant analysis of metabolic differences among adipose tissues from different depots. (**A**) PCA score plot of the four groups; (**B**,**D**,**F**,**H**,**J**,**L**) PLS-DA score plots between two groups; (**C**,**E**,**G**,**I**,**K**,**M**) Permutation test plots for the corresponding PLS-DA models between two groups.

To investigate global metabolic variations among adipose depots, we first performed unsupervised principal component analysis (PCA) to obtain an unbiased overview of inherent metabolic differences (independent of group labels). PCA revealed a clear separation trend between hump adipose tissue (HAT) and mesenteric adipose tissue (MAT) along the PC1 axis (explaining 34% of total variance; Figure 2A), indicating fundamental differences in their metabolic phenotypes. In contrast, perirenal (PRAT) and pericardial (PAT) adipose tissues clustered closely, suggesting high metabolic similarity between these visceral depots. This unsupervised analysis provides the primary evidence for metabolic segregation. To further maximize inter-group separation and identify depot-specific fatty acid signatures, we subsequently employed a supervised partial least squares discriminant analysis (PLS-DA), whose results corroborated the PCA-derived patterns. In pairwise comparisons of HAT with MAT, PAT, and PRAT, all groups exhibited complete inter-group separation (Figure 2B,D,F,H,J,L), underscoring the distinct metabolic specificity of HAT relative to other visceral depots. Notably, no clear separation was observed between PAT and PRAT (Figure 2H,J,L), reinforcing their high metabolic congruence. Given the modest sample size (*n* = 13), we rigorously validated all PLS-DA models to guard against overfitting. Using 7-fold cross-validation optimized for our replicate number, we found all models yielded R^2^X > 0.6, R^2^Y > 0.4, and Q^2^ > 0.2, indicating robust data fitting and generalizability. We further conducted 200-cycle permutation tests to formally assess overfitting risk: all models showed Q^2^ regression line intercepts below zero (Figure 2C,E,G,I,K,M) and achieved permutation *p*-values < 0.05. While no single metric can definitively rule out overfitting, the convergence of these validation results collectively supports the reliability of the PLS-DA models and suggests a low probability of spurious separation. Thus, PLS-DA findings are presented as supplementary evidence to guide differential metabolite identification in subsequent sections, with the core conclusion of depot-specific metabolic segregation remaining rooted in the unbiased PCA results.

### 3.3. Comparative Analysis of Fatty Acid Composition Among Different Adipose Depots

To investigate the differences in fatty acid profiles among adipose tissues from distinct depots of Junggar Bactrian camels, the composition of 40 fatty acids in hump adipose tissue (HAT), mesenteric adipose tissue (MAT), pericardial adipose tissue (PAT), and perirenal adipose tissue (PRAT) was determined using GC-MS. As shown in Table 2, at the level of overall lipid class composition, HAT exhibited a distinct fatty acid profile consistent with its proposed role as a specialized energy reservoir: total saturated fatty acids (SFA) were significantly lower in HAT (55.10% ± 1.97%) than in visceral depots (average 60.88% ± 1.75%), while total monounsaturated fatty acids (MUFA) were significantly higher in HAT (34.60% ± 2.82%) than in visceral depots (average 28.32% ± 1.23%). These class-level shifts exceeded the 10% threshold often used to denote biological relevance in lipidomics. Total polyunsaturated fatty acids (PUFA) were modestly elevated in PAT (5.63% ± 0.59%) relative to MAT (5.01% ± 0.54%, FDR-P < 0.05); however, the 12.4% difference was relatively small and likely represents a limited biological impact.

Among individual fatty acids, only palmitoleic acid (C16:1n-7), met both statistical and biological criteria, its content in HAT (4.22% ± 0.71%) was 1.7-fold higher than the visceral average (2.66% ± 0.28%), suggesting a targeted functional enrichment. In contrast, most statistically significant differences reflected minor quantitative variation unlikely to drive physiological outcomes: stearic acid (C18:0) in HAT (20.24% ± 1.65%) was 24% lower than in MAT (26.62% ± 1.33%), oleic acid (C18:1n-9) in HAT (28.49% ± 2.84%) was 17% higher than the visceral average (24.51% ± 0.73%). Similarly, myristic acid (C14:0, PAT vs. MAT), pentadecanoic acid (C15:0, PAT vs. HAT/MAT), and heptadecanoic acid (C17:0, HAT vs. others) showed statistically significant but negligible (<5%) differences. Palmitic acid (C16:0) was numerically higher in HAT (23.23% ± 0.95%) than in visceral depots (22.85% ± 0.68% average), though this difference met neither statistical nor biological thresholds. The remaining fatty acids showed no consistent depot-specific patterns meeting dual-threshold criteria, with full data provided in Table S1.

### 3.4. Screening and Identification of Differential Metabolites Among Different Adipose Depots

**Figure 3:**
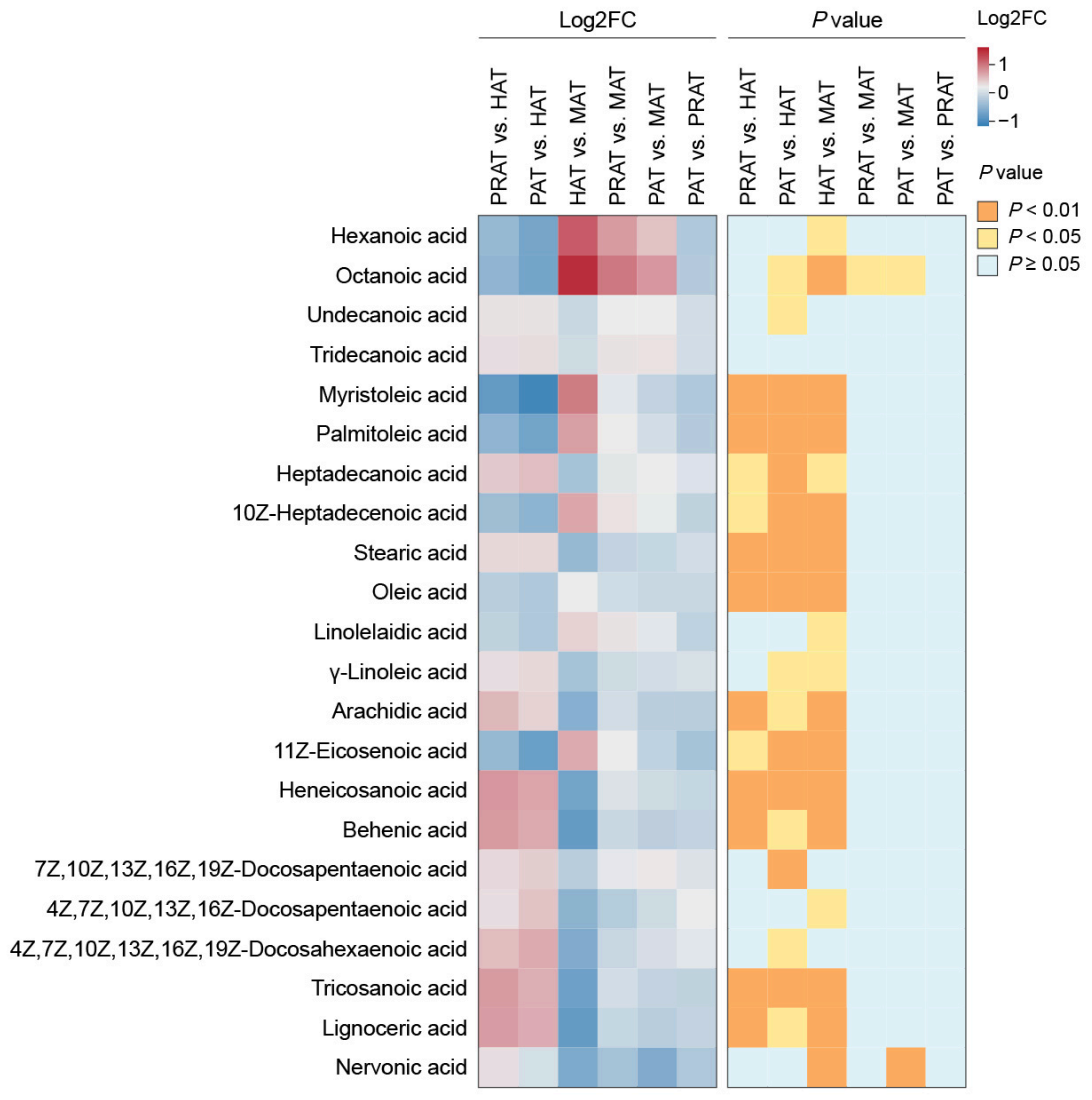
Heatmap visualization of differentially abundant metabolites across four adipose tissue depots. Note: The left panel displays the Log_2_ fold change (Log_2_FC) values, where red indicates upregulation and blue indicates downregulation in the column group compared to the row group (e.g., red in the ‘PAT vs. HAT’ column signifies higher abundance in PAT than in HAT). The right panel shows the statistical significance, with color intensity representing *p*-values (orange for *p* < 0.01; yellow for *p* < 0.05). Metabolites (rows) and comparison groups (columns) were hierarchically clustered using Euclidean distance. Only metabolites meeting the criteria of FDR-adjusted *p* < 0.05 and FC ≥ 1.2 or ≤0.83 are displayed.

Differentially abundant metabolites analysis among hump adipose tissue (HAT), mesenteric adipose tissue (MAT), pericardial adipose tissue (PAT), and perirenal adipose tissue (PRAT) revealed distinct metabolite profiles across depots (Figure 3). A total of 10 differential metabolites, including myristoleic acid, stearic acid, and tricosanoic acid, were identified between PRAT and HAT, of which 6 were upregulated and 4 were downregulated (Table S2). Between PAT and HAT, 11 differential metabolites were identified, including myristoleic acid, palmitoleic acid, and stearic acid, with 5 upregulated and 6 downregulated. Between HAT and MAT, 15 differential metabolites were identified, including stearic acid, oleic acid, and palmitoleic acid, with 8 upregulated and 7 downregulated. No differential metabolites were detected between PRAT and MAT, PAT and MAT, or PAT and PRAT.

### 3.5. Analysis of Key Differential Metabolic Pathways

**Figure 4:**
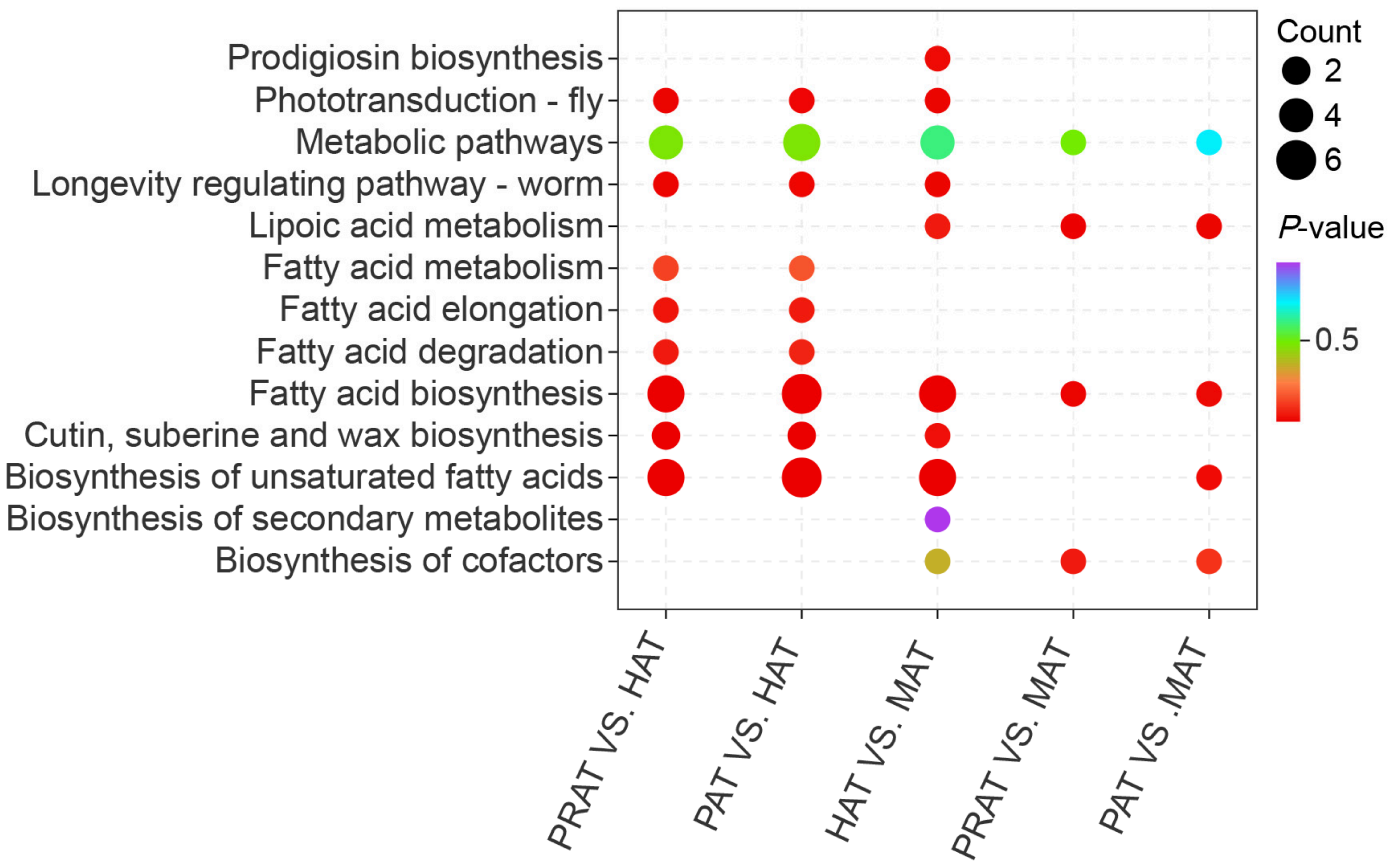
KEGG Enrichment Analysis Diagram.

To further elucidate the metabolic heterogeneity among adipose tissues from different depots of Junggar Bactrian camels, KEGG pathway enrichment analysis was performed on the identified differential metabolites (Figure 4). The results demonstrated that, in pairwise comparisons of hump adipose tissue (HAT) with mesenteric adipose tissue (MAT), pericardial adipose tissue (PAT), and perirenal adipose tissue (PRAT), the fatty acid biosynthesis pathway and the biosynthesis of unsaturated fatty acids pathway were both highly significantly enriched. Aligned with the coordinated shift toward monounsaturated fatty acid accumulation and saturated fatty acid reduction in HAT, these enrichments prompt a hypothesis that fatty acid synthesis and desaturation processes may contribute to the distinct metabolic phenotype of the hump. We emphasize that this inference is derived solely from steady-state metabolite profiling: as this study did not quantify enzymatic activity, gene expression, protein abundance, or metabolic flux of these pathways, we cannot confirm them as causal drivers of HAT specialization, and this proposed link requires validation via future functional assays.

### 3.6. Coupling of Adipocyte Morphology and Fatty Acid Composition Across Adipose Depots

To investigate the association between depot-specific fatty acid profiles and adipocyte morphology, a Mantel test was performed to assess the congruence between metabolic and morphological distance matrices. The analysis revealed a weak but statistically significant positive correlation between the overall fatty acid composition and adipocyte structural features (Mantel’s r = 0.18, *p* = 0.012), suggesting that tissue-specific lipidomic signatures are modestly linked to cell architecture.

**Figure 5:**
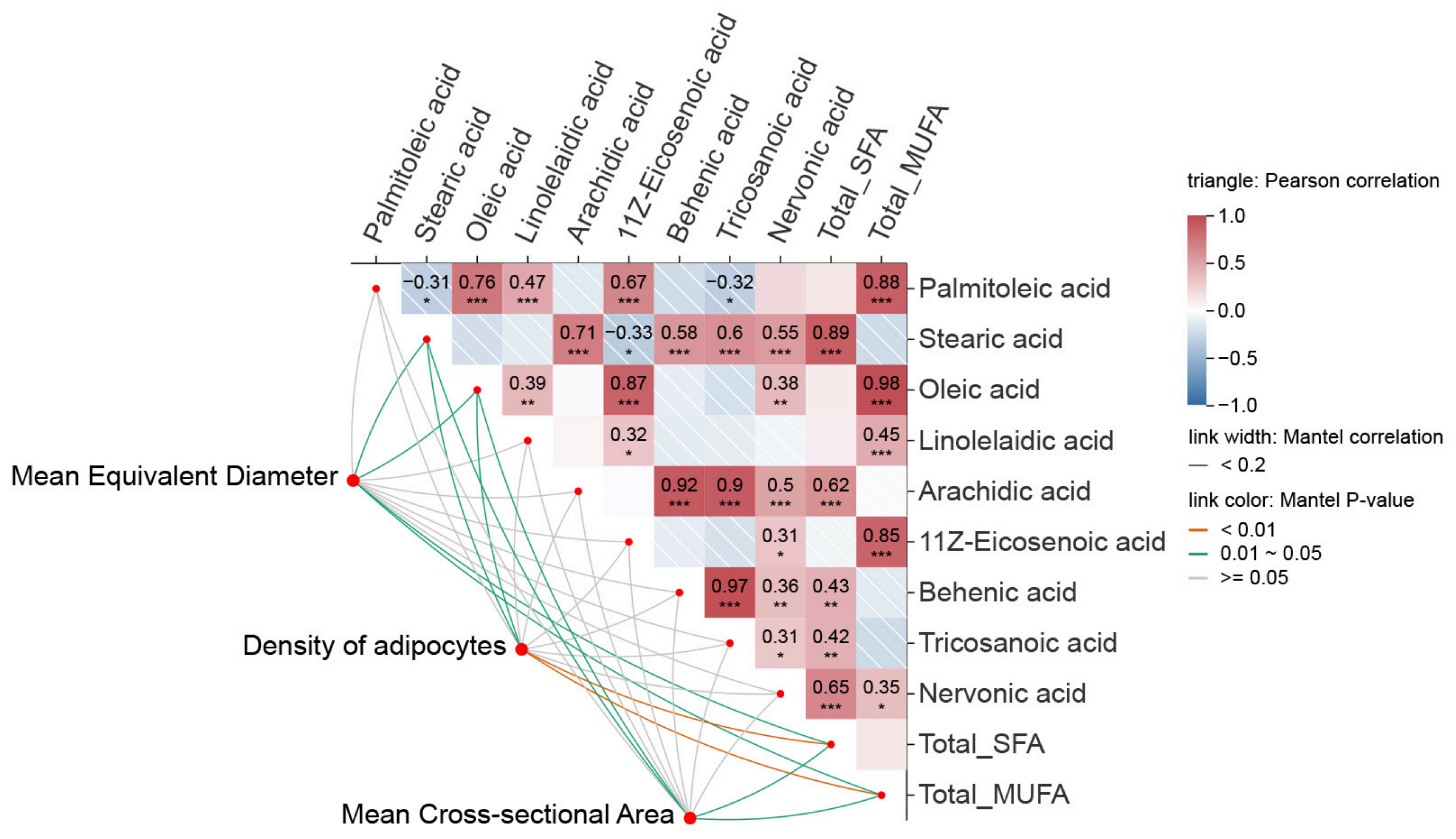
Mantel test correlation heatmap. Note: ***: *p* < 0.001, **: *p* < 0.01; *: *p* < 0.05.

To identify specific metabolites driving this association, we examined Pearson correlations between individual fatty acids and morphological parameters (Figure 5). Due to the large number of metabolites, only those exhibiting significant correlations or representing major lipid classes are displayed for clarity. Total saturated fatty acids (Total_SFA) and total monounsaturated fatty acids (Total_MUFA) showed positive correlations with mean equivalent diameter and cross-sectional area. Among individual fatty acids, palmitoleic acid (C16:1), oleic acid (C18:1), and nervonic acid (C24:1) were positively associated with cell size, whereas arachidonic acid (C20:4) and stearic acid (C18:0) exhibited distinct associations with cell density. The comprehensive correlation matrix detailing all fatty acids is available in Table S3.

## 4. Discussion

As a quintessential desert-adapted species, the camel exhibits a highly anatomically specific distribution and function of adipose tissue [21]. In the present study, systematic comparison of cellular morphological parameters across different adipose depots via H&E staining revealed marked differences in adipocyte size and density among perirenal adipose tissue (PRAT), hump adipose tissue (HAT), pericardial adipose tissue (PAT), and mesenteric adipose tissue (MAT). We observed that PRAT exhibited significantly greater mean equivalent diameter and cross-sectional area than HAT and MAT. Previous studies have established that perirenal adipose tissue, as a major visceral fat depot, possesses a unique anatomical configuration characterized by abundant blood supply, sympathetic innervation, and close proximity to the kidney, conferring upon it a specialized role in energy metabolism distinct from that of subcutaneous and other visceral fat depots [22]. While these morphological disparities provide a structural basis for hypothesized functional specialization, we distinguish these observational findings from functional outcomes; assertions regarding endocrine regulation, energy storage efficiency, or mechanical protection are derived from correlative interpretations within the extant literature rather than direct evidence from this study. Rotondo et al. [23] demonstrated, in Wistar rats, that perirenal adipocytes were significantly larger than subcutaneous adipocytes and exhibited enhanced lipid storage capacity, corroborating the notion that this depot functions as an efficient energy reservoir. Such large adipocyte volume likely enables PRAT to rapidly accommodate substantial lipid loads during periods of energy surplus. In contrast to PRAT, HAT displayed uniformly small adipocytes with exceptionally high lipid droplet occupancy. Histological examination confirmed a dense architectural arrangement within the hump, characterized by tightly packed adipocytes and a homogeneous distribution of unstained cytoplasmic vacuoles. These vacuoles represent processing artifacts resulting from the solvent-mediated extraction of intracellular lipids during H&E staining. Epicardial and pericardial adipose tissues, situated in close apposition to the coronary arteries and myocardium, are widely recognized to serve as local energy buffers and mechanical protectors for the heart [24]. Our findings revealed that PAT exhibited intermediate adipocyte size between HAT and PRAT. Dibouni et al. [25] demonstrated in mouse models that PAT possesses a distinct genetic expression profile and histological characteristics, with an adipokine secretion pattern that differs from those of subcutaneous and gonadal adipose depots. This unique molecular signature likely underpins PAT’s specialized role in cardiac homeostasis. Through systematic comparison of adipocyte morphological parameters across four anatomically distinct depots, our findings establish that cellular morphological divergence constitutes a critical microstructural basis potentially driving functional specialization among fat depots in energy storage, endocrine regulation, and mechanical protection.

Subsequently, targeted metabolomic analysis revealed that hump adipose tissue (HAT) exhibited the greatest number of differentially abundant metabolites (12–18) when compared with the other three depots (PRAT, PAT, and MAT), substantially exceeding the minimal differences observed among visceral depots (PRAT vs. MAT: 1; PAT vs. MAT: 2). Notably, no significant differentially abundant metabolites were detected between PAT and PRAT. This pattern of metabolic heterogeneity aligns closely with the anatomical localization and physiological roles of each depot. Given that HAT is proposed to function as a critical energy reserve enabling camels to survive in arid environments [26], its distinct lipid profile may reflect an integration of metabolic pathways suited for dynamic energy regulation [27]. In contrast, PAT and PRAT, both classified as visceral adipose subtypes, participate cooperatively in the maintenance of systemic energy homeostasis and thus share a highly conserved targeted fatty acid composition. Between HAT and MAT, a total of 18 key differential metabolites were identified, of which 8 were upregulated and 10 were downregulated. Stearic acid, one of the most abundant saturated fatty acids in camel adipose tissue, accounted for 20.24% of total fatty acids in HAT. Unlike most saturated fatty acids, dietary stearic acid does not elevate serum low-density lipoprotein cholesterol levels, exerting a neutral effect similar to that of oleic acid [28]. Prior studies in bovine adipose tissue suggest that stearic acid imposes a lower hepatic burden than other fatty acids during lipolysis [29]; however, we did not directly measure lipolysis, hepatic metabolism, or circulating fatty acids in the present study. In the present study, stearic acid content was lower in HAT but relatively higher in MAT, potentially reflecting divergent lipolytic rates, fatty acid oxidation capacities, and energy supply modalities between these two depots. Oleic acid, the most abundant monounsaturated fatty acid in both HAT and MAT, constituted 24.36% of total fatty acids in HAT. As an Omega-9 monounsaturated fatty acid, exogenous oleic acid has been shown to prevent iron overload toxicity and inhibit ferroptosis in cell culture models; in mouse models, oleic acid protected against FAC-induced hepatic lipid peroxidation and injury [30]. Additionally, Ursula et al. [31] demonstrated that oleic acid limits adipose tissue lipolysis in transition dairy cows, improves systemic and adipose tissue insulin sensitivity, and is associated with mitochondrial function markers that support a shift toward lipogenesis in adipose tissue during the periparturient period. Palmitoleic acid, an Omega-7 monounsaturated fatty acid, was present at lower levels than stearic acid and oleic acid in camel adipose tissue, accounting for 4.22% of total fatty acids in HAT. Emerging evidence indicates that palmitoleic acid functions as a beneficial lipokine released by adipose tissue, protecting against obesity and mitigating the negative effects of excessive circulating non-esterified fatty acids (NEFAs) on systemic glucose metabolism [32]. Susan et al. [33] reported that intravenous infusion of palmitoleic acid in obese sheep significantly reduced weight gain, intramuscular adipocyte size, total lipid content, and circulating insulin levels. Notably, the contents of both oleic acid and palmitoleic acid were significantly higher in HAT than in MAT. This disparity likely reflects the distinct physiological functions and metabolic mechanisms of these two depots. As a specialized energy storage organ unique to camels, HAT supplies energy during prolonged drought and fasting [26]. While studies in other models suggest that oleic acid may preserve stability via antioxidant properties and that palmitoleic acid may function as a lipokine to enhance insulin sensitivity [30,31,32,33], we acknowledge that these mechanisms were not measured in camels in this study. The synergistic interplay between these two fatty acids may collectively contribute to sustaining the structural integrity and functional efficiency of HAT under extreme environmental conditions.

The present study revealed that differential metabolites between HAT and MAT were significantly enriched in fatty acid biosynthesis and biosynthesis of unsaturated fatty acid pathways. The core pathway of fatty acid synthesis initiates with acetyl-CoA, which is carboxylated by acetyl-CoA carboxylase to generate malonyl-CoA. Malonyl-CoA is subsequently converted to palmitic acid by the fatty acid synthase complex. As the primary end product of de novo lipogenesis, palmitic acid can be further elongated to stearic acid by the elongase enzyme ELOVL6 [34,35]. Following the generation of saturated fatty acids, the synthesis of monounsaturated fatty acids is primarily catalyzed by stearoyl-CoA desaturase 1 (SCD1) [36]. SCD1 acts on two substrates: it desaturates palmitic acid (C16:0) to produce palmitoleic acid (C16:1 n-7), and simultaneously desaturates stearic acid (C18:0) to produce oleic acid (C18:1 n-9) [37]. Notably, contrary to our previous assertion, quantitative results demonstrated that HAT exhibited lower total SFA and higher total MUFA compared to visceral depots. In the HAT versus MAT comparison, stearic acid content was significantly downregulated in HAT, while palmitoleic acid and oleic acid contents were significantly upregulated. Given that enzyme expression and activity were not measured in this study, we hypothesize that these compositional shifts may reflect enhanced SCD1-mediated desaturation efficiency in HAT, wherein newly synthesized saturated precursors are rapidly converted to monounsaturated fatty acids rather than accumulating. This pattern leads us to hypothesize that, potentially mediated by SCD1, newly synthesized saturated precursors (C16:0 and C18:0) appear not to accumulate in HAT but rather are preferentially channeled into rapid conversion to monounsaturated fatty acids, serving as efficient metabolic substrates.

Based on Mantel test analysis, the present study constructed a correlation network linking adipocyte morphological parameters with relative fatty acid contents. The results revealed that adipocyte morphology is significantly correlated with fatty acid composition, reflecting a potential depot-specific coordination between tissue microstructure and lipid chemistry. Specifically, mean equivalent diameter and mean cross-sectional area of adipocytes exhibited highly significant positive correlations with very-long-chain fatty acids (VLCFAs). Given their extended hydrophobic carbon chains, VLCFAs possess high melting points and adopt tightly packed molecular arrangements in vitro, which could theoretically enhance lipid droplet stability. In larger adipocytes, VLCFAs may contribute to the structural integrity of lipid stores, aligning with the hypothesized role of visceral depots in long-term energy storage [38]. Furthermore, adipocyte density showed significant positive correlations with specific fatty acid species, such as arachidonic acid and behenic acid. Arachidonic acid serves not only as an energy substrate but also as a precursor for bioactive lipids, including prostaglandins and leukotrienes [39], which are implicated in inflammatory regulation and cellular signal transduction [40]. While the observed positive correlation between cell density and arachidonic acid content does not establish causality, it raises the possibility that denser adipose tissues might be more poised to engage eicosanoid-related signaling pathways. Collectively, this correlation network analysis suggests a potential interplay between the morphological characteristics of adipose tissue and its chemical composition, highlighting the need for future functional studies to elucidate the nature of these associations across camel adipose depots.

While this study provides a foundational atlas of depot-specific morphology and fatty acid composition in the Junggar Bactrian camel, several limitations warrant consideration. Firstly, although we endeavored to maintain uniform housing and dietary conditions (via an intensive fattening system with standardized concentrates and alfalfa hay), environmental and management factors may exert residual influences on adipose tissue plasticity that were not quantified herein. Secondly, sample handling biases represent a potential constraint: while adipose tissues were expeditiously harvested and either fixed or snap-frozen, the inevitable interval between exsanguination and tissue collection, coupled with the solvent-mediated extraction of intracellular lipids during hematoxylin and eosin (H&E) staining, may introduce minor artifacts in cellular morphology. Finally, our investigation focused exclusively on fatty acid profiling; the absence of complementary proteomic or transcriptomic data limits our capacity to mechanistically substantiate the inferred metabolic pathways. Future multi-omics studies integrating functional assays are required to validate these correlative findings and elucidate the causal regulatory networks governing adipose specialization in camels.

## 5. Conclusions

By integrating histomorphological analysis with GC-MS-based targeted fatty acid profiling, this study delineates pronounced differences in adipocyte morphology and fatty acid composition among adipose depots in the Junggar Bactrian camel. Hump adipose tissue (HAT) exhibited small, densely packed adipocytes and significantly higher levels of palmitoleic acid (C16:1 n-7) and oleic acid (C18:1 n-9), defining a distinct biochemical signature relative to visceral depots. Perirenal adipose tissue exhibited the largest adipocyte diameters, while visceral depots generally contained higher proportions of saturated fatty acids. Correlative analyses suggest potential links between these compositional differences and depot-specific physiological roles, such as energy storage or structural support, which warrant future experimental validation. Pathway enrichment analysis identified fatty acid biosynthesis as the primary pathway associated with these metabolic differences. These findings provide a foundational atlas of adipose depot specialization in the Junggar Bactrian camel, laying the groundwork for future investigations into the physiological and applied significance of these depots.

## Figures and Tables

**Table 1:** Morphometric characteristics of adipose depots in Junggar Bactrian camels.

Parameter	MAT	HAT	PRAT	PAT
Mean Equivalent Diameter (μm)	60.51 ± 14.591 ^A^	69.57 ± 13.479 ^B^	77.21 ± 18.831 ^C^	74.88 ± 15.387 ^C^
Mean Cross-sectional Area (mm^2^)	0.0033 ± 0.0014 ^A^	0.0044 ± 0.0015 ^B^	0.0055 ± 0.0023 ^C^	0.0050 ± 0.0017 ^C^
Adipocyte Density (cells/mm^2^)	407.08 ± 268.928 ^A^	286.89 ± 198.719 ^B^	246.32 ± 174.946 ^B^	255.78 ± 176.102 ^B^

Note: Data are expressed as mean ± SD (*n* = 13). Different uppercase letters (A, B, C) within a row indicate significant differences (FDR-adjusted *p* < 0.05) based on RM ANOVA with Tukey’s post hoc test.

**Table 2:** Comparison of relative fatty acid content (%) among different adipose depots.

Fatty Acid	PAT	PRAT	HAT	MAT
Myristic acid (C14:0)	7.83 ± 0.724 ^a^	8.21 ± 0.653 ^ab^	8.30 ± 0.441 ^ab^	8.44 ± 0.624 ^c^
Pentadecanoic acid (C15:0)	1.33 ± 0.345 ^a^	1.23 ± 0.367 ^ab^	1.03 ± 0.343 ^b^	1.03 ± 0.211 ^b^
Palmitic acid (C16:0)	22.83 ± 1.085	22.72 ± 1.185	23.23 ± 0.954	22.99 ± 0.901
Heptadecanoic acid (C17:0)	1.99 ± 0.311 ^a^	1.82 ± 0.362 ^ab^	1.38 ± 0.257 ^c^	1.67 ± 0.231 ^b^
Stearic acid (C18:0)	25.79 ± 2.351 ^ab^	24.90 ± 1.142 ^b^	20.24 ± 1.648 ^c^	26.62 ± 1.327 ^a^
Palmitoleic acid (C16:1n-7)	2.58 ± 0.541 ^b^	2.94 ± 0.301 ^b^	4.22 ± 0.713 ^a^	2.47 ± 0.557 ^b^
Elaidic acid (C18:1 trans-9)	0.63 ± 0.107	0.63 ± 0.139	0.55 ± 0.119	0.54 ± 0.069
Oleic acid (C18:1n-9)	23.81 ± 2.305 ^b^	24.37 ± 1.842 ^b^	28.49 ± 2.837 ^a^	24.36 ± 1.199 ^b^
Linoleic acid (C18:2n-6)	3.19 ± 0.444	3.14 ± 0.566	2.87 ± 0.598	2.81 ± 0.381
α-Linolenic acid (C18:3n-3)	1.69 ± 0.289	1.65 ± 0.318	1.54 ± 0.296	1.53 ± 0.211
Total SFA (SFA)	60.84 ± 2.959 ^ab^	60.00 ± 1.834 ^b^	55.10 ± 1.972 ^c^	61.81 ± 1.458 ^a^
Total MUFA (MUFA)	27.89 ± 2.625 ^b^	28.91 ± 1.899 ^b^	34.60 ± 2.821 ^a^	28.17 ± 1.460 ^b^
Total PUFA (PUFA)	5.63 ± 0.587 ^a^	5.55 ± 0.812 ^ab^	5.15 ± 0.845 ^ab^	5.01 ± 0.536 ^b^

Note: Data are expressed as mean ± SD (*n* = 13), calculated by normalizing individual fatty acid concentrations to the sum of all quantified FAMEs. Different uppercase letters (a, b, c) within a row indicate significant differences (FDR-adjusted *p* < 0.05) based on one-way repeated-measures ANOVA with Tukey’s post hoc test, performed on these normalized percentage values.

## Data Availability

The metabolomics data supporting the findings of this study are openly available in the figshare repository at https://doi.org/10.6084/m9.figshare.32648607.

## Supplementary Materials

The following supporting information can be downloaded at https://www.mdpi.com/article/10.3390/ani16152412/s1, The relative molar percentages of fatty acids (Table S1); Absolute Quantification and Methodological Validation (Table S2); Comprehensive correlation matrix between fatty acid profiles and adipocyte morphological parameters (Table S3).

## Abbreviations

MAT	Mesenteric adipose tissue
HAT	Hump adipose tissue
PRAT	Perirenal adipose tissue
PAT	Pericardial adipose tissue
QC	Quality control
EI	Electron ionization
